# Emerging Role of Arbuscular Mycorrhizal Fungi in Sustainable Agriculture: From Biology to Field Application

**DOI:** 10.1002/mbo3.70082

**Published:** 2025-10-23

**Authors:** Ajay Kumar, Reetesh Kumar, Pallavi Singh, Susmitha Kalaichelvan, Sergio de los Santos‐Villalobos, Naveen Kumar, Luiz Fernando, Rajeev Kumar, Manoj Kumar Solanki, Naveen Chandra Joshi, Olubukola Oluranti Babalola

**Affiliations:** ^1^ Amity Institute of Biotechnology Amity University Noida Uttar Pradesh India; ^2^ Department of Biotechnology & Bioengineering, School of Biosciences and Technology Galgotias University, Greater Noida Uttar Pradesh India; ^3^ Department of Biotechnology Graphic Era (Deemed to be University) Clement Town Dehradun Uttarakhand India; ^4^ Centre for Herbal Pharmacology and Environmental Sustainability, Chettinad Hospital and Research Institute Chettinad Academy of Research and Education Kelambakkam Tamil Nadu India; ^5^ Instituto Tecnológico de Sonora Ciudad Obregón Sonora Mexico; ^6^ Department of Physics Galgotias University Greater Noida India; ^7^ Graduate Program in Genomic Sciences and Biotechnology Catholic University of Brasília Brasília Brazil; ^8^ Department of Life Sciences and Biological Sciences IES University Bhopal Madhya Pradesh India; ^9^ Amity Institute of Microbial Biotechnology Amity University Noida India; ^10^ Food Security and Safety Focus Area, Faculty of Natural and Agricultural Sciences North‐West University Mmabatho South Africa; ^11^ Berkshire UK

**Keywords:** arbuscular mycorrhizal fungi (AMF), commercial production, substrate‐free approach, in vitro approach, stress management, symbiosis

## Abstract

In recent years, increasing consumer demand for organic food and chemical free agricultural products has driven a shift toward microbial‐based approaches, which are being adopted to replace traditional agrochemicals, used for nutrient supplementation and protection against plant pathogens. Arbuscular mycorrhizal fungi (AMF) can form symbiotic associations with up to 80% of plant roots, are widely employed as bio stimulants, biofertilizers, or biopesticides to improve agricultural productivity. Currently, a range of AMF strains are commercially produced and applied as soil inoculants to improve agricultural yields. Although the effectiveness of these inoculants depends on multiple factors, including the selection of AMF strains, choice of carrier materials and methods of application. In addition, production strategies play a critical role in determining both the concentration and the viability of the inoculum. Despite significant technological advancements, only a limited number of AMF strains have been commercially exploited as inoculants. Thus, the present review aims to briefly discuss the latest aspects of AMF biology, their functional role in abiotic and biotic stress management. Furthermore, this review paper also discusses different production strategies and highlights the challenges associated with the commercialization of AMF inoculants, including limited strain diversity, propagule viability, formulation stability, and inconsistent field performance.

## Graphical abstract

Explore the molecular mechanism of AMF and Plant symbiosis. Briefly discussed the AMF mediated biotic and abiotic stress management. The review explores the latest production strategies and application challenges of AMF. Discuss how the commercial success of AMF inoculants depends upon several factors

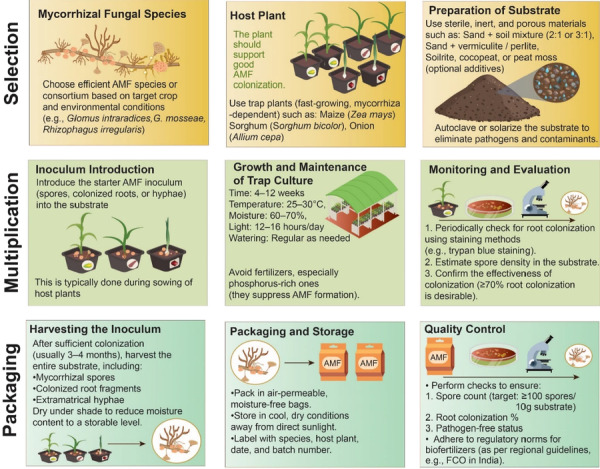

**Abbreviations**
ABA: abscisic acid
CAT: catalase
ET: ethylene
ETI: effector‐triggered immunity
GSH: glutathione
ISR: induced systemic resistance
JA: jasmonic acid
MAMPs: microbe‐associated molecular patterns
MTI: MAMP‐triggered immunity
POD: peroxidase
PRR: pattern recognition receptor
ROS: reactive oxygen species
SA: salicylic acid
SAR: systemic acquired resistance
SOD: superoxide dismutase

## 1 Introduction

In the current era of changing global climate and limited land resources, ensuring food security is a significant challenge. Moreover, crop yield losses caused by abiotic stresses, plant diseases, or phytopathogen attacks during growth or storage further compound these challenges. As per the report of FAO 2009, “How to feed the world in 2050” the projected global population by 2050 is estimated to reach around 10 billion, which required extra 70% food from the current levels to ensure food security for all (Hossain et al. 2024). However, over the last two decades, despite improvements in the agricultural yield, abiotic stress such as salinity, drought, variation in soil pH, or biotic stresses caused by pest or pathogens have resulted in significant agricultural loss (Igiehon 2019).

Ensuring healthy plant growth requires protecting crops from different stresses through sustainable practices, so that their nutritional needs are met and optimal yields can be achieved (Babalola et al. 2021). However, a significant proportion of farmers worldwide particularly in the developing countries, predominantly relies on traditional methods, such as chemical fertilizers to meet nutrients demands and chemical pesticides for plant disease management (Chen et al. 2023). However, the continuous and indiscriminate use of agrochemicals harms soil properties, fruit quality, and native soil microbial communities. Additionally, it also causes soil or environmental pollution, because studies showed that only 1% of the applied agrochemicals reach the target sites, a large proportion leach out to the soil and water ecosystem and cause pollution (Mostafalou and Abdollahi 2012; Mubeen et al. 2023; Porrini et al. 2003; Rathore and Nollet 2012). Over the past two decades, new methods have been explored to enhance crop yields and protect plants from various stressors (Van Driesche et al. 2010; Begum et al. 2019). The application of beneficial microbial strains to improve crop yields or reduce biotic and abiotic stresses represents a developing, eco‐friendly, and cost‐effective strategy (Igiehon and Babalola 2021).

Plant hosts a diverse array of microbial communities, including bacteria, fungi, nematodes, and protozoans, some of which play crucial roles in enhancing growth and maintaining plant stability against stressful conditions. Arbuscular mycorrhizal fungi (AMF) form symbiotic associations with plant roots and provide multiple benefits to their host plants. These benefits include enhancing nutrients acquisition, particularly phosphorus and micronutrients, improved water uptake, and increased tolerance to abiotic stresses such as drought, salinity, and extreme soil pH. In return, host plants supply carbohydrates to the AMF (Bothe 2012; Köhl et al. 2016). Additionally, AMF contribute significantly to soil health by improving soil structure, facilitating nutrient cycling, and supporting the broader microbial community. Notably, AMF have also been reported to reduce the emission of the potent greenhouse gas nitrous oxide (N₂O), highlighting their potential role in climate‐smart agriculture (Bender et al. 2014).

Plant‐AMF association plays crucial role in overall growth of plants even under stress conditions (Berdeni et al. 2018; Krishna et al. 2010; Wu et al. 2013; Begum et al. 2019; Igiehon and Babalola 2021; Sonbol et al. 2025). For instance, Mathur et al. (2019) observed AMF inoculation protects the photosynthetic machinery of wheat under drought conditions. In another study, Abdel Latef and Chaoxing (2011) demonstrated that AMF enhances growth performance and antioxidant enzyme in tomato under saline condition. Similarly for biotic stress management, Berdeni et al. (2018) reported inoculation AMF reduce the disease incidence of *Dematophora necatrix* and *Botryosphaeria* sp. in young apple trees and enhances the nutrient acquisition capacity. Krishna et al. (2010) reported that AMF inoculation suppresses *Botryosphaeria* canker of apple.

Therefore, this review aims to briefly discuss the recent advances in the biology of AMF, emphasizing their functional roles in mitigating abiotic stresses and biotic stresses. Furthermore, it discusses current strategies for AMF inoculant production and the challenges associated with their commercial application in field conditions.

## 2 An Overview of AMF Biology

The historical record of AMF starts with the 480 million years ago, co‐evolved with the plants (Redecker et al. 2000; Delaeter et al. 2024) and currently reported in about 80% of the total plant species of various families or genera. To date, more than 352 species of AMF are reported and Glomeromycetes are the most dominant group, classified into five orders (Paraglomerales, Archaeosporales, Entrophosporales, Glomerales, and Diversisporales as per recent AMF classification of Wijayawardene et al. (2022). AMF constitute a major part of the soil microbiome, contributing to over 50% of the total microbial biomass (Olsson et al. 1999; Smith and Read 2008; Köhl et al. 2016). AMF presence in the soil mediates various functions; however, their diversity can be reduced after intensive use of agrochemicals, tillage practices and the cropping of non‐host crops (Säle et al. 2015). Although study also showed that reduction of AMF diversity adversely affects ecosystem services (Wagg et al. 2011).

**Figure 1:**
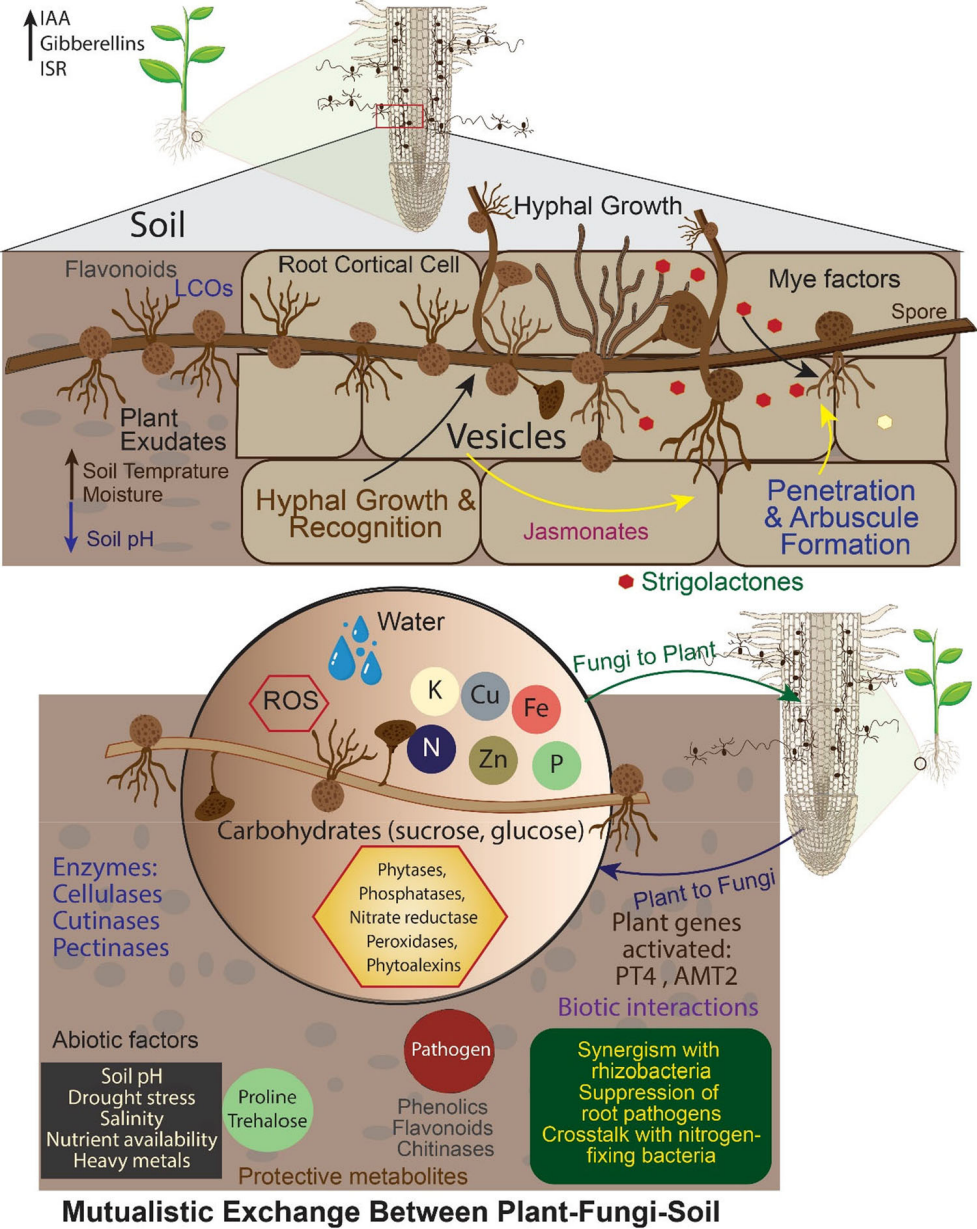
Mycorrhizal Symbiosis: growth stages, mechanisms, and environmental interactions in plant roots. This figure shows the sequential steps of AMF colonization of plant roots, from spore germination to mature nutrient exchange. It emphasizes important signaling molecules, e.g. strigolactones, Mycorrhizal Lipo chito oligosaccharides (LCOs), enzymatic processes (e.g., cellulases, phosphatases), and plant responses (e.g., increased root growth, stress tolerance). Multiple interactions show bidirectional nutrient exchange, root gene expression, and ecological functions, such as enhanced soil structure and decreased fertilizer requirement. Environmental (pH, drought, salinity) and biotic (rhizobacteria, pathogens) interactions are incorporated. Induced Systemic Resistance (ISR); Arbuscular mycorrhizal fungi (AMF); ROS (Reactive Oxygen Species); Indole‐acetic acid (IAA); PT4 (phosphate transporter); AMT2 (ammonium transporter).

AMF are obligate biotrophs that cannot independently mineralize organic nutrients in the soil due to their limited exoenzymatic capabilities (Bonfante and Perotto 1995). As a result, they rely on mutualistic associations with host plants to acquire essential nutrient (Steinkellner et al. 2007; Tisserant et al. 2013; Reinhart et al. 2012). AMF depend on plant roots to complete their life cycle as symbiotic organisms, with their primary role being the supply of nutrients and water to the host. The extraradical hyphae of AMF, which extend beyond phosphorus‐deficient zones in the soil, are particularly important for providing the host plants with phosphorus (P), an essential macronutrient (Reinhart et al. 2024) (Figure 1). This extended hyphal network not only facilitates the efficient absorption of phosphorus but also improves the uptake of other immobile nutrients such as zinc (Zn) and copper (Cu). In addition to nutrient acquisition, AMF hyphae contribute to improved soil structure by promoting aggregation through the production of glomalin, which enhances water retention and aeration. Through these mechanisms, AMF play a critical role in optimizing plant nutrient status, mitigating abiotic stresses such as drought and salinity, and fostering overall soil health (Ryan et al. 2012; Begum et al. 2019).

The symbiotic interaction between AMF and the host plant begins when AMF hyphae reach the root surface. In response, the root epidermal cells undergo structural modifications to form a pre‐penetration apparatus, which facilitates the entry of the AMF intra‐radical mycelium into the host roots through the intercellular spaces of the cortical cells (Delaeter et al. 2024). This intra‐radical mycelium, after reaching the host cells, creates branching structures called arbuscules, which mediate nutrient acquisition (Harrison 2012; Gao et al. 2021). However extra‐radical mycelia exhibit a network structure and are distributed across the soil. Extraradical mycelia can penetrate sites inaccessible to plant roots and mediate nutrient exchange (van der Heijden et al. 2015; Schouteden et al. 2015; Xie et al. 2022). Although their branching and growth are influenced by modifications in AMF metabolism, this process leads to the development of a new main mycelium (Vierheilig et al. 1998). However, to establish and sustain a successful symbiotic relationship, AMF must locate a suitable host plant capable of consistently supplying them with energy‐rich photosynthetic products (Rouphael et al. 2015). Remarkably, before these partners physically interact or communicate, they start to transmit signalling molecules. For example, host root cells release strigolactones, which are sensed by AMF and speed up the branching of their hyphae.

The AMF is more likely to approach the plant roots closely as a result of this increased branching (Gholamhoseini et al. 2013). But the AMF's signal, which is commonly referred to as “myc” factors, is detected by the host root and enriched with Ca2^+^, which in turn triggers the “sym” pathway. This results in modifications to the morphology, gene expression, and physiological functions of the plant (Uhe 2018). These adjustments are designed to provide ample space for AMF and to make it easier for the synergistic association to function harmoniously. But according to a prior study, AMF strains suppress the immune system by secreting specific protein or signalling molecules that aid in colonization of the host plants (Kloppholz et al. 2011).

Additionally At the genetic level, effective colonization by AMF involves the sensing and recruitment of various biochemical compounds, including fatty acids, sugars, and carotenoids (van der Heijden et al. 2015; Rouphael et al. 2015; An et al. 2019). The process depends upon two cassette transporters namely ATP–binding cassette or RAM2 (required for Arbuscular mycorrhization2) exchange of fatty acids (Jiang et al. 2017; Wang et al. 2024). The glycerol‐3‐phosphate acyltransferase RAM2, responsible for transporting lipids from plants to AMF, is regulated by the transcription factor RAM1, which is essential for establishing AM symbiosis. Lipid biosynthesis enzymes such as FatM and the ABC transporter STR are also critical during the early phases of symbiosis and have been specifically retained in plants that engage in AM symbiosis (Bravo et al. 2017; Wang et al. 2024). Sugars serve as another critical component, providing energy and carbon skeletons to sustain fungal growth. Studies in *Medicago truncatula* have demonstrated that efficient sugar transport from host plants is essential for maintaining symbiotic functionality and promoting arbuscule development (An et al. 2019).

## 3 Significance of AMF in Abiotic Stress (Salinity and Drought) Management

**Figure 2:**
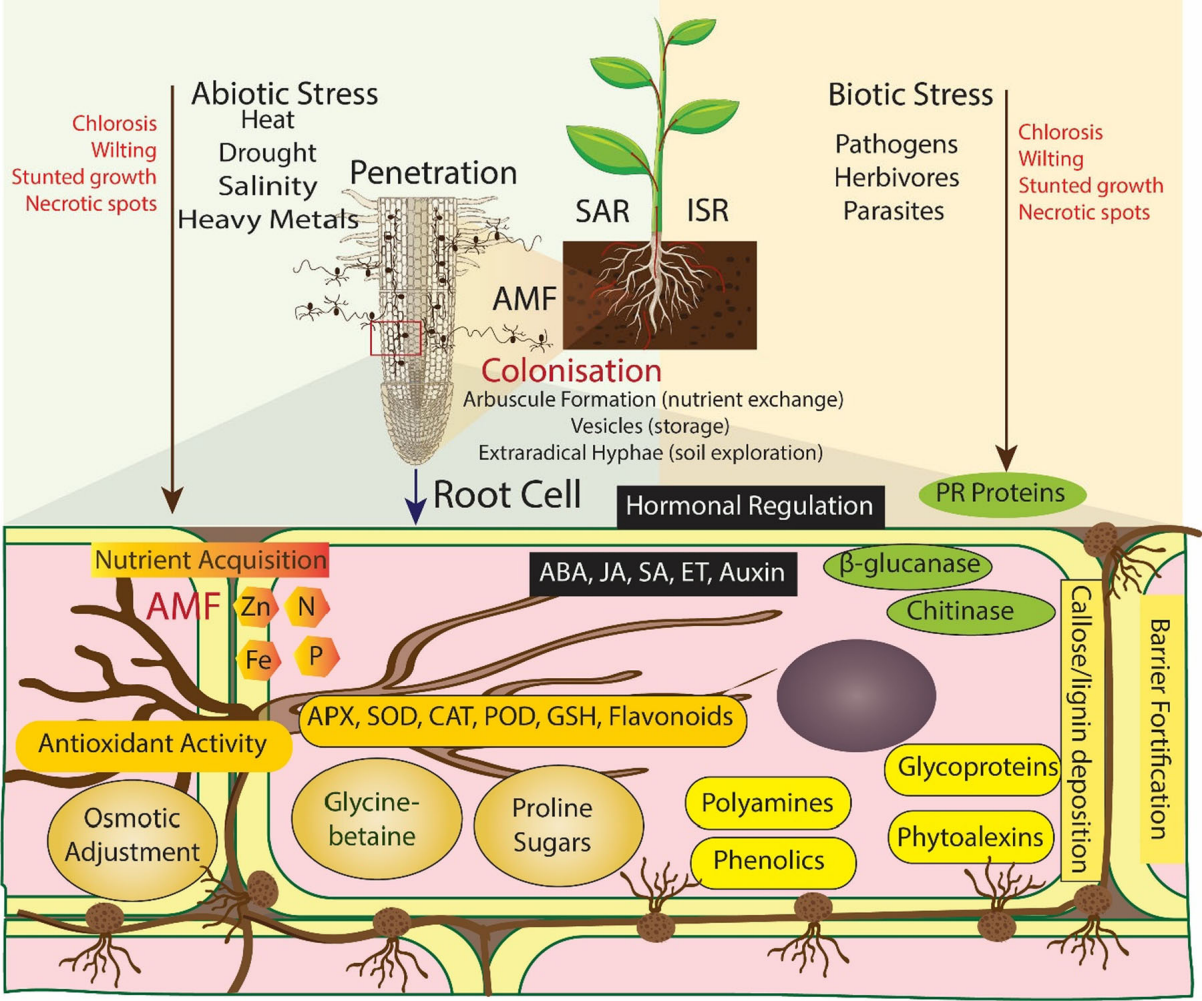
AMF‐mediated plant stress tolerance mechanisms. This diagram illustrates how AMF enhance plant resilience to abiotic and biotic stresses through diverse interactions within the plant rhizosphere. Abiotic and biotic stresses induced symptoms such as chlorosis, wilting, stunted growth, and necrotic spots. AMF colonization facilitates nutrient exchange via arbuscule formation and vesicular storage, while extraradical hyphae enhance soil exploration. This AMF symbiosis activates systemic immunity in plants via SAR (systemic acquired resistance) and ISR (induced systemic resistance) pathways, which crucially help in improving plant defence system. The SAR triggered in plants by the pathogen infection, however ISR triggered by the beneficial microorganisms including AMF. Within the plant cell, AMF influences hormonal regulation, nutrient acquisition and osmotic adjustment via accumulation of glycine‐betaine, proline, sugars, and polyamines. Antioxidant activity is facilitated by the antioxidative enzymes which mitigate oxidative stress. However, defensive metabolites are upregulated, callose deposition and lignin fortification strengthen the barrier against pathogens. PR (Pathogenesis‐Related) proteins further enhance immunity, collectively enabling plants to withstand diverse environmental challenges through AMF‐mediated mechanisms.

In the current context of shifting climatic conditions, abiotic stressors such as drought, salt, and pH fluctuations exacerbate food security issues (Igiehon and Babalola 2018a; Igiehon and Babalola 2018b). At present, agricultural practices in many regions particularly in developing countries still rely heavily on conventional methods, such as rain‐fed irrigation and the use of chemical fertilizers. However, over the past two decades, the rise in greenhouse gas emissions and shifting climatic patterns have contributed to increased environmental temperatures, erratic seasonal changes, and a decline in overall precipitation. All these factors affect agricultural productivity and also limit water availability due to drought stress conditions. Similar to this, the increasing rate of deforestation enhances the rate of desertification, resulting in salinity stress, which also affects agricultural productivity. The prolonged stress condition may cause the death of plants. The abiotic stressors affect plant growth and development due to impairment in the plant physiological or metabolic processes (Boutasknit et al. 2021) (Figure 2).

Plant development is significantly impacted by drought stress because it changes the permeability of membranes and lowers transpiration rate, which affects the intake of water and nutrients respiration, photosynthesis, translocation of photo‐assimilates, plant growth, and regulators of plant development and nutritional metabolism. Net photosynthesis in plants can be decreased by stomatal closure caused by decreasement in the underground water (Goicoechea et al. 2005). Additionally, drought can lead to generation of Reactive Oxygen Species (ROS). ROS are the highly reactive oxygen species produced during normal cellular metabolism and stress conditions. However, the excess accumulation affects the cellular integrity or can cause denaturation of proteins or nucleic acids (Impa et al. 2012; Begum et al. 2019).

Salinity stress primarily results from the substantial accumulation of Na^+^ in the soil surrounding the plant rhizosphere or within plant cells. Plants with an elevated Na^+^ ion concentration face challenges or an inability to absorb other vital ions, such as K^+^, Ca^2+^, and Zn^2+^, which are essential for regulating plant physiological and metabolic processes (Begum et al. 2019; Ahanger et al. 2018). Osmotic stress and ion toxicity are the principal mechanisms through which salinity stress negatively affects plant growth and survival (Ahanger et al. 2018). Osmotic stress is the result of the accumulation of a high saline concentration around the plant root cells, resulting in a higher water potential than that of the soil solution. The insufficient ability of the roots to absorb water causes a physiological water deficit in plants, resulting in stomatal closure, which reduces CO_2_ uptake and, consequently, photosynthesis (Qin and Huang, 2020). However, the plant system tries to maintain osmotic adjustment via osmoregulation and the production of osmolyte compounds, like proline and malondialdehyde (MDA), for the proper cellular development, expansion, and water absorption (Begum et al. 2019). Dehydrated plants experience alterations in intercellular osmotic pressure and reduced nutrient transport efficiency, leading to nutritional deficiencies (Chen et al. 2020). Like drought stress salinity also releases different types of ROS on the advent of salinity stress, which disrupt cell membrane integrity and also damage the nucleic acid (Ahanger et al. 2018; Soussani et al. 2025). Therefore, to manage the ROS, plants secrete antioxidative enzymes like SOD, CAT, POD, which neutralize the activity of ROS. AMF symbiosis helps in neutralizing the ROS by modulating the concentration of antioxidative enzymes (Chu et al. 2008; Delaeter et al. 2024). Previously various authors reported such types of observation. For example, Thangaraj et al. (2022) reported inoculation of *Glomus mosseae* in *Sorghum biocolor* enhanced SOD, POD, CAT, proline and GSH activities in compared to non‐inoculated control seedling under the drought condition. Similarly, Abdelaal et al. (2024) reported inoculation of AMF strain in the wheat plant significantly increased antioxidative enzymes proline content and decreased the concentration of H_2_O_2_, O^2−^ and MDA content under drought condition.

### 3.1 AMF Enhance the Drought Resistance of Host Plants

Drought stress significantly affects the primary metabolism of plants, as stomatal closure a key mechanism to reduce water loss limits CO₂ availability, thereby impairing photosynthesis and ultimately reducing growth and crop yields. Drought initiates an imbalance in the photosystem, resulting in the accumulation of reactive oxygen species, lipid peroxidation, and chlorophyll degradation (Ruiz‐Sánchez et al. 2010; Boutasknit et al. 2021). Nevertheless, focusing on the symbiotic relationship between plants and AMF, various strategies have been explored to mitigate drought stress (Igiehon and Babalola 2018a; Igiehon and Babalola 2017). The fungi in question are recognized for their capacity to generate a glomalin‐like soil protein, essential for augmenting soil carbon sequestration and enhancing soil aggregate stability. The AMF symbiosis enhanced the plant root growth by influencing auxin production and transport. The AMFs are associated with soil characteristics that enhance porosity, thereby optimizing plant‐water dynamics and facilitating nutrient absorption (Al‐Karaki et al. 2004; Begum et al. 2019; Bahadur et al. 2019) increased leaf area index, chlorophyll content or photosynthetic pigments (Begum et al. 2019), by synthesizing antioxidative enzymes (Impa et al. 2012), by regulating stomata conduction, by modulating phytohormonal level, especially abscisic acid concentration (Duan et al. 1996).

The water transport system in plants is severely impacted by the expression patterns of specific drought‐related genes, including plasma membrane intrinsic proteins (PIPs), Rir‐AQP1, and Rir‐AQP2, which are aquaporins (AQPs) that regulate water movement across cell membranes. Huang et al. (2020a) noted increased concentrations of aquaporins, specifically PIPs, in *Malus hupehensis* seedlings inoculated with AMF under drought stress, in contrast to non‐inoculated controls. Similarly, Li et al. (2013) reported a significant upregulation of the aquaporin genes in AMF‐colonized plants exposed to drought stress, indicating the role of AMF in enhancing water transport and drought tolerance. Previously, authors reported different AMF strains that were successfully implemented for drought stress management. Wu et al. (2022) highlighted the significance of AMF in agriculture, particularly in rain‐dependent regions, where water availability is a limiting factor for crop productivity. AMF inoculation has been shown to enhance both shoot and root biomass; for example, one study reported a 23% increase in total biomass of N₂‐fixing plants and a 29.6% increase on average root biomass compared to non‐inoculated controls under drought condition. Li et al. (2025) also observed similar results in maize after treatment with AMF strain *Funneliformis mosseae*. However, Cui et al. (2024) observed drought tolerance in sugar beet after AMF inoculation. Ye et al. (2023) observed AMF significantly enhanced drought tolerance in *Vitis vinifera*, by regulating osmotic balance or enhancing the expression of drought responsive genes. Hu et al. (2020) reported enhanced drought tolerance by altering photosystem II efficiency, chlorophyll content, soluble sugars, GABA concentration and regulating concentration of some other metabolites in Maize after AMF inoculation. In a study Wu and Xia (2006) reported improved osmotic regulation, photosynthesis, plant growth or biomass in *Citrus tangerine* after inoculation of *Glomus versiforme* AMF strain. Chen et al. (2025) reported that inoculation of AMF maintains the rhizosphere microbiome composition of maize that help in maintaining growth and stability under drought condition. Shang et al. (2025) reported AMF inoculation significantly improved the drought stress in walnut by improving photosynthetic and ROS scavenging ability. The AMF inoculation enhanced the SOD, peroxidase and catalase activity by 19.9%, 18.43% and 8.39%. in the walnut.

### 3.2 Role of AMF in Salinity Stress Management

Salinity stress adversely impacts plant growth and development due to the toxic effects of Na^+^ and Cl^–^, which interfere with plant functions, and the reduction in water availability around the roots (Wang et al. 2024). However, to alleviate these effects, plants have evolved various mechanisms, including signal transduction pathways, such as protein kinases, phosphatidylinositol, and calcium signalling, as well as the modulation of phytohormone levels. This results in adaptive reactions, including the creation of compatible solutes, ion efflux, and the regulation of ROS homeostasis, among others. Additionally, plants can develop symbiotic associations with beneficial microbial species, including AMF, to combat the adverse impact of salinity stress (Evelin et al. 2019). For example, Hamzehzadeh et al.(2025) reported AMF inoculation improve the photosynthetic efficacy and optimized ionic ratio or enhanced K, Ca, Mg, P in the pistachio plants under salinity stress condition. Cimen et al. (2025) reported enhanced fresh weight, chlorophyll content and photosystem II efficacy in *Diospyros lotus* after AMF inoculation.

Plant with AMF exhibit a lower concentration of soluble sugar and Na^+^ when exposed to salt stress compared to non‐mycorrhizal halophyte seedlings. These variations may be connected to the regulation of energy and carbohydrate metabolism, such as the metabolic pathways for glyoxylate and dicarboxylic acid (Diao et al. 2021). Additionally, AMF inoculation has been reported to enhance chlorophyll content and improve light energy utilization, thereby protecting plants from salinity stress, as demonstrated in *Phoebe zhennan* (Cui et al. 2021). Previously, authors reported enhanced pattern of salt tolerance genes like OsPRX, Os10g, and *OsHBP1b*, in the AMF inoculated plants. This enhanced concentration of genes regulated ROS scavenging ability and decreased the MDA contents in the plants. Zhang et al. (2023) reported enhanced expression of salt tolerant genes in rice. Wang et al. (2022a) reported enhanced expression of salt stress tolerance genes HAK5, PIP1‐2, MYB46, NAC43 in *C. glauca* after inoculation with *R. irregularis*. In a study Pooja et al. (2024) reported improved morphological yields like length of root and shoots, nodules number and biochemical components like proline, flavonoids, anthocyanin content in *Cicer arietinum* L. after inoculation of *Rhizophagus fasciculatus* under salinity stress condition. Similarly, Pu et al. (2025) reported enhancement in salinity tolerance in soyabean after exogenous treatment of AMF. The application of AMF significantly lowered Na^+^ ion or MDA content and reduced CAT activity. Yuan et al. (2024) reported salinity tolerance in *Cannabis sativa* and Kakabouki et al. (2023) in flax after AMF treatments.

## 4 Role of AMF in Biotic Stress Management

Currently biotic stress caused by various phytopathogen such as bacteria, fungi, nematodes lead to significant losses in crop yields. AMF inoculation has been shown to significantly reduced pathogen invasion and manage plant diseases at different stages of plant growth through multiple direct or indirect mechanisms (Hashem et al. 2025; Sonbol et al. 2025). These mechanisms include competition for nutrient and space, modification in root morphology, and the activation of plant defence system (Boyno et al. 2025). An overview of AMF mediated biotic stress management presented in Figure 2.

### 4.1 Competition for the Nutrient and Space

The competition for nutrient acquisition in resource‐limited environments creates intense interactions between AMF and plant pathogens (Ismail et al. 2013; Hennecke et al. 2023). In symbiotic associations, AMF strains obtain photosynthetic products from the host plant for sustenance and growth, while pathogenic microorganisms similarly exploit these photosynthates upon infection. This overlap generates a competitive dynamic in which AMF can indirectly suppress pathogen development by limiting the availability of host‐derived nutrient. In addition, AMF can occupy root colonization sites, reducing the space available for pathogen entry, and can induce systemic resistance in the host plant by activating defense‐related pathways, including the production of pathogenesis‐related proteins, phytohormones such as salicylic acid and jasmonic acid, and secondary metabolites. These mechanisms enable AMF to mitigate the effects of biotic stress, enhancing plant health and productivity in environments prone to pathogen pressure. (Wheatley and Poole 2018; Igiehon and Babalola 2017).

### 4.2 The Alteration in the Rhizospheric Interactions

AMF also control rhizospheric interactions that enhance the disease resistance of plants. AMF improves the nutrient uptake, especially phosphorus, by the establishment of large hyphal networks, which improves the health and immunity of plants (Igiehon and Babalola 2017). Gao et al. (2020) reported that the AMF strain *Rhizophagus irregularis* enhanced nutrient uptake, particularly phosphate utilization, in cotton plants. Fungal hyphae also form physical barriers around the roots that resist the entry and colonization of pathogens in the plants. AMF modulate the native microbial structure of the host plant by favouring the growth of beneficial microorganisms (Igiehon et al. 2021). Additionally, release of bioactive compounds modulates root exudation patterns, favouring beneficial fungi and bacteria that lead to disease suppression (D'ALESSANDRO et al. 2014; Cameron et al. 2013). In a study, Wang et al. (2021) reported modulation in the population dynamics of rhizobia in *Medicago truncatula* rhizosphere after AMF inoculation, which leads to encouragement in the nodulation process and ultimately improve the plant performance. Yang et al. (2024) reported *R. intraradices* treatment enhanced population dynamics of rhizospheric strain *Pseudomonas psychrotolerans* having ability of N2‐fixation and also regulate the metabolic pathways related with plant resistance against the stress condition. Wen et al. (2023) reported *R. intraradices*, inoculation enhance the population of *Talaromyces verruculosus* responsible for phosphate uptake and reduce the abundance pathogenic strain *Nigrospora oryzae*.

### 4.3 Activation of Plant Defences System During Phytopathogen Invasion

The AMF‐mediated plant defense system involves various factors, including chemical signaling, receptor recognition, and immune responses. Although AMF employ several mechanisms to suppress phytopathogens, activation of the plant's defense system is one of the most prominent strategies. It is well established that both AMF and pathogens possess specific surface molecules referred as microbe‐associated molecular patterns (MAMPs), which are recognized by plant receptors to initiate defense signaling. However, to sense these MAMPs, plant cells have PRR proteins that allow them to identify these surface chemicals. The identification of MAMPs and PPRs initiates the immune responses through either effector‐triggered immunity (ETI) or MAMP‐triggered immunity (MTI) (Kashyap et al. 2023), leading to programmed cell death (Mur et al. 2008). AMF constituents, such as ergosterol and chitin, also function as MAMPs and trigger the plant immune system (Felix et al. 1993).

Plants can detect these inducers and have robust defence mechanisms to respond to pathogen assaults. These active defence mechanisms entail the secretion of diverse enzymes and proteins, including phytoalexins, chitinases, and β−1,3‐glucanases, which are crucial in suppressing phytopathogens (Kumari et al. 2022). Previous studies have shown that inoculation with the AMF *Funneliformis mosseae* on tomato plants infected with *Alternaria solani* resulted in a significant reduction in disease incidence. A significant enhancement in defence‐related enzyme activity was noted in the treated tomato leaves (Song et al. 2015). An analogous increase in defence enzymes was observed in wheat plants following AMF inoculation (Mustafa et al. 2017).

The AMF also protect the pathogen invasion via inducing SAR or ISR. In the SAR, AMF colonization triggers the synthesis of pathogenesis‐related (PR) proteins, which contribute to enhanced plant immunity (Liu 1995) and mediated via modulation and cross talk of salicylic acid signalling molecules. However, in the ISR, modify the cell wall structure via deposition of callose around the hyphae that inhibits the colonization of pathogenic microorganisms and mediated via the modulation of jasmonic acid or ethylene level (Jung et al. 2012). Additionally, AMF colonization induces a priming response, which prepares the plant for a faster and stronger activation of defense mechanisms upon pathogen attack. This primed response enables plants to react more rapidly compared to non‐primed individuals during pathogen invasion (Jung et al. 2012).

### 4.4 Role of AMF in Regulating Morphology of Plant Roots

The root colonization efficacy of the AMF lead to their impact on root branching or regulation of root morphological attributes. This regulation in root morphology may be due to modulation in phytohormone or direct impact of AMF which ultimately lead to enhancement in nutrient or minerals uptake, incensement in water absorption area (Fusconi 2014). Even so, the effects of AMF inoculation change according to the form of root; for example, tap roots have better absorption potential of water and minerals than fibrous roots (Yang et al. 2015; Huang et al. 2020b). During pathogen attack, pathogens typically attempt to penetrate plant cells through the cell wall. However, AMF symbiosis strengthens the host plant's physical barriers by inducing lignification, thickening of root epidermal cells, and modifying root morphology. These changes collectively enhance cell wall integrity and restrict pathogen entry into the roots (Boutaj et al. 2019). In a study, Pozo et al. (2002) reported the restriction of pathogen *Phytophthora infection* in the AMF inoculated tomato plants and found that AMF inoculation led to modification in epidermis or endodermis of roots by the callose or pectin molecules, which prevent the entry of pathogen into the host root tissues.

## 5 AMF as Microbial Inoculants: Current Strategies

Microbial inoculants are cultures of beneficial microbes, either in solid, liquid, or granular form, used to treat plants in vitro or *in‐ vivo* for sustainable agricultural practices. Similarly, AMF inoculants are formulated from single AMF strains or consortia of two or more strains, and can also include mixtures with other beneficial microbes, such as bacteria or different fungal groups, to enhance plant growth and productivity in sustainable farming systems (Alladi et al. 2017; Singh et al. 2021; Kamath et al. 2023).

**Figure 3:**
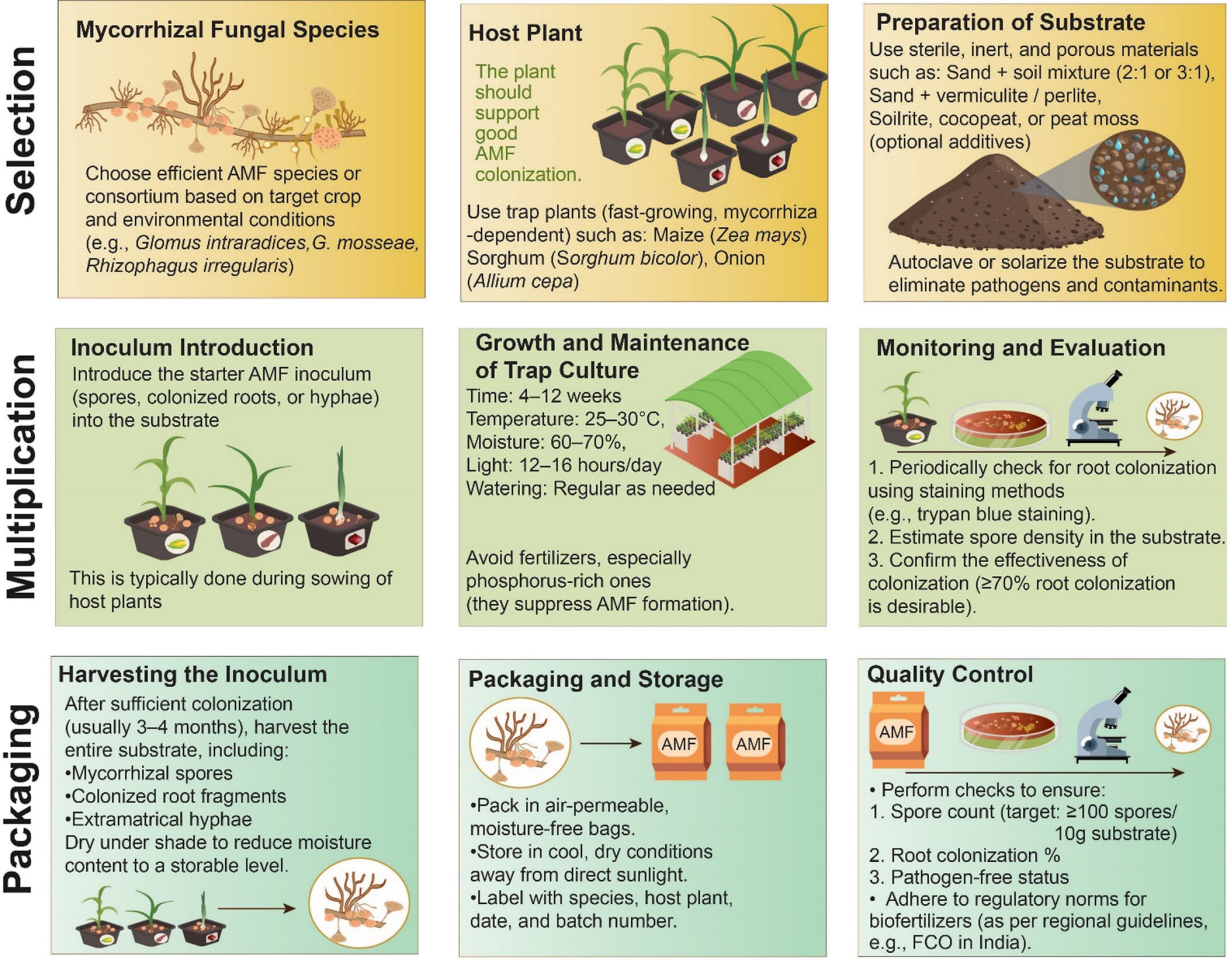
Workflow for arbuscular mycorrhizal fungi (AMF) Inoculum Production and Application. This schematic outlines the steps for AMF inoculum production: Selection of AMF species (e.g., *Glomus intraradices*, *G. mosseae*, *Rhizophagus irregularis*) based on target crop and environment; Host Plant selection (e.g. maize sorghum, onion for AMF colonization; Preparation of Substrate using sterile sand, soil, vermiculite, perlite, or peat moss (2:1 or 3:1 ratio), autoclaved or solarized; Inoculum introduction of AMF spores, colonized roots, or hyphae during sowing; Growth and maintenance in trap culture (25°C–30°C, 60%–70% moisture, 12–16 h light, regular watering, avoiding phosphorus‐rich fertilizers); Monitoring and evaluation via staining (e.g. trypan blue), spore density estimation, and confirming ≥ 70% root colonization; Harvesting after 3–4 months, collecting spores, roots, and hyphae, followed by drying; Packaging and Storage in air‐permeable, moisture‐free bags, avoiding sunlight, with labeling; and Quality Control ensuring ≥ 100 spores/10 g substrate, ≥ 70% root colonization, and pathogen‐free status, adhering to regional biofertilizer norms (e.g., FCO in India).

However, the formulation of AMF inoculants involves a series of steps, and for successful commercial product, specific quality standards must be maintained throughout the process, from the selection of AMF strains to packaging and field application (Benitez et al. 2016; Barazetti et al. 2019) (Figure 3). However, it has been also observed that not all the crops, soils or the production system positively respond after AMF inoculation and in several cases other alternative approaches like microbial or agronomic interventions, biochar application found more precise and effective (Hart et al. 2018; Mason et al. 2025; Lahijanian et al.^2025^).

### 5.1 Selection of the AMF Strains and Viability

Selection of appropriate strains is the primary requirement for formulating an AMF bioinoculant. This selected AMF strain should possess strong colonization potential across a broad range of host plants. Previous studies have reported that most broad commercial AMF inoculants belong to the *Rhizophagu*s sp. or *Glomus* sp. Selected strains should also exhibit genetic stability under varying environmental conditions (Rojas‐Sánchez et al. 2022) and showed better ability to survive under local field conditions such as soil pH, temperature, and a within the native soil microbiota (Chaudhary et al. 2024; Rúa et al. 2016; Singh et al. 2020; Santoyo et al. 2021).

Long‐term viability and a high density of viable propagules are essential for optimal colonization of host plants. Another critical consideration is that the propagules must be free from contamination. During commercial production, AMF spores are often produced in vivo by inoculating host plants such as maize or sorghum, which naturally harbor a diverse range of microorganisms. Consequently, it is necessary to screen AMF inoculants for potential pathogenic contaminants to ensure product safety and efficacy (Salomon et al. 2022). However, a number of studies showed that some plant species, especially non‐mycorrhizal crops or those in heavily fertilized soils, can exhibit little or even adverse reactions to AMF inoculation. The detection of such unresponsive systems is important to prevent unnecessary input expenditures for farmers and to be evidence‐based in recommendations (Ryan and Graham 2018; Kumar et al. 2019; Sharma et al. 2023).

### 5.2 Adjuvants/Adhesives or Carriers

In the formulation of AMF, adjuvants or adhesives or carriers are employed to facilitates inoculum adhesion, however for the commercial purposes carriers must possess some special attributes like assure microbial stability, substantial moisture retention capacity, ease of processing, sterilization convenience, sufficient seed adhesion, affordability, and inhibit dispersion during application or sowing (Kumar et al. 2022; Poppeliers et al. 2023). Additional aspects, including the potential for interaction with other substances, such as nutrients and adjuvants, must also be considered (Malusá et al. 2012; Barazetti et al. 2019).

Adjuvants often consist of natural or synthetic polymers, polysaccharides, or polyalcohol derivatives. Each adjuvant has unique characteristics; for example, CMC (Carboxymethyl cellulose), frequently employed as an adjuvant, can prolong shelf life (Zhou et al. 2017). PVP protects microorganisms against dehydration and toxins (Singleton et al. 2002); starch molecules improve the colonization efficacy (Bashan et al. 2002; Ghorui et al. 2025). The effective distribution of AMF propagules within a suitable carrier medium is essential for ensuring the efficacy and viability of the inoculant. Various carrier systems have been developed for agricultural applications and environmental remediation, including algal or polymer‐based beads (Vassilev et al. 2005), liquid formulations (Malusá and Vassilev 2014), biochar substrates (Sashidhar et al. 2020), and seed coatings (Rocha et al. 2019). Coarse materials like processed clay are also used to enhance the manageability and application of the inoculants (Vassilev et al. 2005). Importantly, the carrier medium must be homogeneous to ensure uniform dispersion of AMF propagules (Salomon et al. 2022).

### 5.3 Selection of Delivery Method

The delivery of inoculants to target sites, such as agricultural fields, is an important aspect, and the delivery agents vary depending on the nature or state of the bioformulation and the strains of the AMF inoculants to ensure practical application. The application method of the bioformulation varies according to the application strategies. For example, the delivery agent used for seed application differs from that used for soil inoculation. However, the key factor for a successful delivery agent is to maintain the uniformity of bioinoculants or bioformulations after application and to ensure optimal interaction with plant roots, thereby enhancing efficacy (Thirumal 2017).

### 5.4 Quality Control (QC)

To ensure the efficacy and safety of AMF bio formulations and to establish trust among the farmers and consumers, quality control is a crucial aspect of assessment. Before the product is properly labelled and marketed, QC begins with an evaluation of the viability of AMF propagules and their colonizing efficacy into the host plant species under controlled conditions. During the QC assay, the viability of the strains has been found effective if it showed growth response in at least 20% colonized roots. The assessment also provides details of the purity of AMF inoculants by identifying any contamination present (Salomon et al. 2022; Ghorui et al. 2025). However, on the laboratory scale, some of the tests, like the growth of microorganisms after inoculating a Petri dish with PDA media, are used to confirm the purity of the flask inoculum. Further random samples will also be analysed microscopically to check the purity of AMF inoculants or commercial fertilizers (Volpato 2020).

### 5.5 Production Strategies of AMF Inoculants

Currently, various strategies are employed to produce large quantities of commercial AMF inoculants, with three primary approaches being widely adopted: substrate‐based production systems, substrate‐free systems, and in vitro cultivation techniques (Wipf et al. 2019; Agnihotri et al. 2024). Each of these methods has specific advantages or disadvantages, which are discussed below.

#### 5.5.1 Substrate‐Based Production System

It is a conventional method, in which sand, soil, and other advanced substrate‐based media have been used for the production of AMF inoculants. Such technologies are extensively utilized and constitute a cost‐effective strategy. In this method, AMF strain is grown in the trap culture, in which the substrate, such as soil, sand or other organic materials, is combined with pieces of roots along with sterilized diluents. After the growth of host plants, it promotes sporulation of AMF by providing a favourable environment and a strong symbiotic relationship. However, it is labour‐intensive and highly prone to contamination, due to the presence of microbial communities in the host plant tissues (Sakha et al. 2024).

To address contemporary challenges related to contamination and efficacy, various synthetic substrates, including perlite, zeolite, vermiculite, and biochar, have been employed. These substrates mitigate contamination risks and provide numerous benefits, such as enhanced nutrient absorption, improved water retention, effective spore dispersal, and enhanced colonization efficacy of AMF inoculants with the host roots (Ijdo et al. 2011; Hu et al. 2022; Papafotiou et al. 2021; Azimi et al. 2019). However, for production, growth chambers should preferably be controlled systems that facilitate or monitor the humidity and temperature within the growth chamber (Gaur and Adholeya 2002). The availability of micro or macro‐nutrients in the medium also affects the large‐scale production of the AMF inoculants. Although a substrate‐based production system is cost‐effective, high chance of contamination is one of its significant disadvantages.

#### 5.5.2 Substrate‐Free Production System

Substrate‐free systems represent a crucial approach for the efficient and scalable production of high‐quality AMF inoculum under controlled, sterile conditions. In these systems, it is essential to ensure that both the seedlings commonly wheat and linseed and the AMF propagules typically belong to group *Glomus intraradices, Acaulospora laevis, Entrophospora kentinensis* are free from contamination before inoculation. Before introduction into the substrate‐free system, seedlings are pre‐cultured in experimental pots containing inert substrates such as sand or perlite, with the pots shielded from light to inhibit algal growth (Ijdo et al. 2011).

Currently, various substrate‐free systems, including hydroponics and aeroponics, are employed to generate contamination‐free, high‐density infective propagules. These approaches, differ primely in the modes of aeration and nutrient solutions delivery. In static systems, where the nutrient solution remains stationary, proper aeration using an aeration pump is essential to prevent oxygen deficiency in the roots. Adequate oxygenation supports the healthy development of extraradical hyphae, which are critical for effective AMF propagation (Dewir et al.^2023^; Vassilev et al. 2017). Substrate‐free systems possess several advantages over traditional soil‐based propagation, including precise control over nutrient availability, reduced possibility of contamination, and potential for high‐volume production of very infective propagules.

#### 5.5.3 *In‐Vitro* cultivation systems

The in vitro cultivation of AMF is well suited for the development of high‐value crops and is generally performed on the excised roots, commonly referred to as “root organ cultures” (ROC). This process helps in the generation of contamination free AMF propagules. In this method, AMF spores and the colonised root fragments are co‐cultivated with *Agrobacterium rhizogenes* root‐inducing (Ri) T‐DNA transformed hairy roots, which facilitate root tissue transformation. Following this, the cultures are maintained in sterile growth environments on synthetic substrates devoid of a host plant (Ijdo et al. 2011; Agnihotri et al. 2024). However for the in vitro cultivation, the most commonly used roots are those of carrot transformed with Ri T‐DNA (Bidondo et al. 2012).

ROC provides a sterile and efficient method for the large‐scale production of AMF inoculum. Compared to conventional mass production techniques, ROC offers several advantages, including higher spore and propagule yields within a shorter time frame, reduced spatial requirements and minimize contamination risks (Marysol et al. 2025). Previously, various authors have demonstrated the efficacy of ROC techniques to produce a large amount of AMF spores. For example, Wood et al. (1985) reported high yields of *Rhizophagus* and *Gigaspora* strains using ROC with *Trifolium incarnatum* and *Arachis hupogea*. Declerck et al. (2001) reported 280,000 spores per litter of *Rhizophagus* and *Daucus carota* achieved per litre using ROC in solid medium. Earlier, Mosse and Hepper successfully cultivated AMF by using excised part of tomato roots and red clover on a gelled medium. Ellatif et al. (2019) enhanced the growth of *Gigaspora gigantean* using tomato ROC supplemented with phenolic compounds. Additionally, besides the high yields of propagules or spores ROC system enable precise control over the nutritional or environmental conditions, which play crucial role in optimization of AMF development and their symbiotic potential. The ROC system represents a highly promising platform for both research and commercial‐scale production of high‐quality, standardized AMF inocula (Ghorui et al. 2025).

## 6 Current Status of Commercial AMF Products

An assessment of the AMF‐based biofertilizer market reveals that the North American sector is anticipated to dominate, propelled by the rising demand for organic products, enhanced acceptance of biofertilizers among rural agriculturists, and the extensive implementation of contemporary irrigation methods, including sprinkler and drip systems for fertigation. Previously, Basiru et al. (2021) briefly discussed different mycorrhizae‐based biofertilizers company, product brand names and the AMF species which are used for the formulation. They reported that the most commonly used AMF species in commercial biofertilizers includes *Rhizophagus iranicus*, *R. irregularis*, *R. aggregatum*, and *Funneliformis mosseae*. During formulation, either single strains or consortia of two or more AMF species are frequently employed, often combined with other beneficial microbial groups, such as fungi (*Trichoderma* spp.) or bacterial strains (*Bacillus* sp., *Azospirillum brasilense*, or *Bradyrhizobium japonicum*). These commercial AMF products are typically applied through seed treatment, soil amendment, or fertilization. Soil treatment aimed to improve soil structure, drainage, and overall health, while fertilization concentrates on supplying essential nutrients to plants.

The current global market for commercial AMF inoculants is estimated to reach approximately 995 million USD (Mordor Intelligence 2024). These commercial AMF products are distributed by numerous companies, across different regions of the world. The demand of the commercial AMF inoculants has steadily increased in last two decades, however many products fail to meet advertised standards or deliver the promised benefits. Major issues observed in these commercial products include nonviable strains, crop mortality, contamination by fungal pathogens, or mislabelling of products (Koziol et al. 2025; Koziol et al. 2025).

For instance, Koziol et al. (2025) reported the trial results of 250 commercial AMF products and 28 laboratory grown inoculants on seven crops. They found significant variability among products, with only one in nine commercial formulations demonstrating a potent ability to enhance plant growth and ensure sufficient colonization by AM hyphae. This highlights the urgent need for quality improvement and maintenance of propagule viability in commercial AMF products. In a meta‐analysis, Wu et al. (2022) reported that AMF inoculation significantly enhanced stress tolerance, crop yields (23%), shoot and root biomass, and pod numbers per plant in thirteen popular crops under rainfed conditions. Similarly, Xu (2024) reported significant enhancement in yield up to 24.2%, protein contents, nutrient contents, especially phosphorus or zinc, spike per plant after AMF inoculation in the wheat crop.

Despite these benefits, most commercial AMF inoculants fail to deliver consistent field performance. In a meta‐analysis Koziol et al. (2025) evaluated the progress of commercial AMF inoculants and reported that the major limitation is the loss of propagule viability by the time the products reach farmers. Less, than < 5% commercial products demonstrated effective root colonization in field trials (Salomon and Watts‐Williams 2025). Several studies have highlighted inconsistent crop responses to commercial AMF inoculants. For example, Faye et al. (2013) reported that seven out of twelve commercial AMF inoculants showed enhanced root colonized efficacy, while only three inoculants were able to increase the maize biomass. Similar types of observation were reported in legume‐flax rotation after commercial AMF Inoculation. The author observed a slight impact on lentil yield, while no effect was recorded on flax crop (Li et al. 2019). Berdeja et al. (2025) reported that inoculation of commercial bioinoculants containing AMF improve the colonization efficacy in grapevine roots, however not found any significant enhancement in AMF population or any differences in the microbial composition. Similarly, in early research Thomsen et al. (2021) reported failure of commercial AMF inoculants in improving performance of vineyard.

## 7 Challenges of Commercial AMF Inoculants

The mass production of the AMF for commercial purposes includes both in‐vivo and in‐vitro approaches. In the global market, AMF fertilizers are generally traded in two forms, powdery form (60%) is the most commonly found formulation; however, liquid formulation covers approximately 29% of total traded AMF fertilizers (Basiru et al. 2021).

The conventional approach of powdery form AMF inoculants follows an in vivo approach, in which cocultivation of AMF strains and host plants in the presence of inert solid substances has been practised to allow propagation. At the end of the cycle, propagule‐reach substrates are harvested (Kumar et al. 2017; Basiru et al. 2021). Although this method is cost‐effective, it carries a high risk of contamination, which remains a significant limitation. Another challenge associated with solid‐substrate production is the prolonged spore dormancy, which can delay the attainment of desired propagule density and limit the release of glomalin, a glycoprotein critical for soil aggregation and structure (Miguel et al. 2007).

In contrast, liquid‐substrate systems address many of these limitations. Liquid inoculants reduce spore dormancy periods, facilitate faster achievement of target propagule densities, and stimulate enhanced glomalin production, which supports soil health. Additionally, liquid formulations are more compatible with fertigation and irrigation systems, allowing for easier and more uniform application in commercial agriculture. However, the relatively short shelf‐life of liquid inoculants remains a challenge for large‐scale commercialization, necessitating further improvements in formulation and storage technologies to ensure stability and efficacy (Calvet et al. 2013; Basiru et al. 2021).

## 8 Conclusion

In recent years, AMF strains have been widely employed as soil inoculants to support sustainable agricultural practices. Among the diverse microbial inoculants available, AMF are gaining increasing attention due to their multifaceted benefits—including enhanced plant growth and fitness, improved nutrient uptake and water absorption, activation of defense‐related gene expression, increased resilience to biotic and abiotic stresses, and greater carbon sequestration. In the last two decades, the various types of commercial AMF inoculants have been used to enhance agricultural yields. These commercial AMF strains are used as soil inoculants in either solid, liquid or granular form and are broadly traded in different parts of the world. However, a very low percentage of these commercial inoculants showed a modulatory effect in the field trial. The major challenges of current commercial AMF products are the viability of propagules. Therefore, to maintain the viability of commercial AMF strains and prevent their decline, there is an urgent need to improve the storage conditions, selection of the best carrier materials or modifications in the production strategies. Another important aspect of commercial AMF products is the use of a minimal number of AMF species. Despite significant advancements in technology, very few AMF species are commercially exploited.

Therefore, extensive research is needed to identify the new strains for the commercialization.

Additionally, understanding the inoculation potential of spores, compared to infected root pieces and hyphal fragments, is crucial for enhancing the quality of AMF biofertilizers. It is crucial to evaluate potential risks associated with AMF biofertilizers, including the possible displacement of indigenous AMF communities and the unexpected introduction of infections. This requires conducting genetic tests on inoculated agricultural soils and the commercial AMF inoculum to evaluate their compatibility, potential risks, and ensure biosecurity.

Although the increasing uncertainty or discrepancies among the farmers particularly due to the uncertain return on investment (ROI), after the AMF inoculants fail to deliver the promises or benefits. It is very crucial to determine the specific crop varieties, soil types, and management regimes in which AMF inoculants perform optimally. Another important aspect is the understanding of the circumstances in which complementary or alternative interventions (e.g., phosphorus‐solubilizing bacteria, salt‐tolerant varieties, or management practices such as cover cropping to stimulate indigenous AMF) may prove more reliable.

## Author Contributions

**Ajay Kumar:** conceptualization, supervision. **Reetesh Kumar:** literature survey, curation. **Pallavi Singh:** literature survey, curation. **Susmitha Kalaichelvan:** literature survey, curation. **Sergio de los Santos‐Villalobos:** literature survey, curation, writing – editing, preparation of final draft. **Naveen Kumar:** literature survey, curation. **Luiz Fernando:** writing – editing, preparation of final draft. **Rajeev Kumar:** writing – editing, preparation of final draft. **Manoj Kumar Solanki:** literature survey, curation, writing – editing, preparation of final draft. **Naveen Chandra Joshi:** writing – editing, preparation of final draft. **Olubukola Oluranti Babalola:** conceptualization, supervision, literature survey, curation, writing – editing, preparation of final draft.

## Ethics Statement

The authors have nothing to report.

## Conflicts of Interest

The authors declare no conflicts of interest.
